# Correlations between Transmembrane 4 L6 Family Member 5 (TM4SF5), CD151, and CD63 in Liver Fibrotic Phenotypes and Hepatic Migration and Invasive Capacities

**DOI:** 10.1371/journal.pone.0102817

**Published:** 2014-07-17

**Authors:** Minkyung Kang, Jihye Ryu, Doohyung Lee, Mi-Sook Lee, Hye-Jin Kim, Seo Hee Nam, Haeng Eun Song, Jungeun Choi, Gyu-Ho Lee, Tai Young Kim, Hansoo Lee, Sang Jick Kim, Sang-Kyu Ye, Semi Kim, Jung Weon Lee

**Affiliations:** 1 Department of Biomedical Sciences, College of Medicine, Seoul National University, Seoul, Republic of Korea; 2 Department of Pharmacy, Research Institute of Pharmaceutical Sciences, Medicinal Bioconvergence Research Center, Tumor Microenvironment Global Core Research Center, College of Pharmacy, Seoul, Republic of Korea; 3 Interdisciplinary Program in Genetic Engineering, Seoul National University, Seoul, Republic of Korea; 4 Department of Biological Sciences, Kangwon National University, Chunchon, Kangwon-do, Republic of Korea; 5 Therapeutic Antibody Research Center, Korea Research Institute of Bioscience and Biotechnology, Daejon, Republic of Korea; SAINT LOUIS UNIVERSITY, United States of America

## Abstract

Transmembrane 4 L6 family member 5 (TM4SF5) is overexpressed during CCl_4_-mediated murine liver fibrosis and in human hepatocellular carcinomas. The tetraspanins form tetraspanin-enriched microdomains (TEMs) consisting of large membrane protein complexes on the cell surface. Thus, TM4SF5 may be involved in the signal coordination that controls liver malignancy. We investigated the relationship between TM4SF5-positive TEMs with liver fibrosis and tumorigenesis, using normal Chang hepatocytes that lack TM4SF5 expression and chronically TGFβ1-treated Chang cells that express TM4SF5. TM4SF5 expression is positively correlated with tumorigenic CD151 expression, but is negatively correlated with tumor-suppressive CD63 expression in mouse fibrotic and human hepatic carcinoma tissues, indicating cooperative roles of the tetraspanins in liver malignancies. Although CD151 did not control the expression of TM4SF5, TM4SF5 appeared to control the expression levels of CD151 and CD63. TM4SF5 interacted with CD151, and caused the internalization of CD63 from the cell surface into late lysosomal membranes, presumably leading to terminating the tumor-suppressive functions of CD63. TM4SF5 could overcome the tumorigenic effects of CD151, especially cell migration and extracellular matrix (ECM)-degradation. Taken together, TM4SF5 appears to play a role in liver malignancy by controlling the levels of tetraspanins on the cell surface, and could provide a promising therapeutic target for the treatment of liver malignancies.

## Introduction

The plasma membrane is structurally important for signal transduction between the intracellular and extracellular environments. A diverse set of membrane proteins with specific membrane domains facilitates this signal transduction [1]. In addition to lipid rafts, which are small, dynamic, and heterogeneous membrane compartments enriched with sterol- and sphingolipids [2], tetraspanin-enriched microdomains (TEMs) are independent organizations of large protein complexes that include tetraspanins, integrins, and growth factor receptors contribute to adhesion, proliferation, and migration [3].

Tetraspanins are linked to the progression of a variety of cancers [4]. Currently, 33 mammalian tetraspanins (TM4SFs) have been identified. These proteins weigh between 20 and 30 kDa and have variable sequence homology. However, all these proteins contain four common transmembrane domains, two cytosolic tails, a short extracellular loop (SEL), and a long extracellular loop (LEL) [1]. CD151 (Tspan24) was first identified as a promoter of metastasis [5]; its expression is increased in liver cancer, compared to normal cells [6]. CD151 functions in cellular migration, invasion, angiogenesis, and drug resistance by forming protein complexes with integrins [4], [7], [8].

CD63 (Tspan30) is a tumor suppressor expressed in endosomes and lysosomes and on the cell surface [9]. The trafficking of CD63 between the cell surface and the internal membranes occurs via AP2, clathrin-coated pit-mediated endocytosis, or caveolae-mediated endocytosis, and it requires specific amino acid motifs present in the CD63 protein [9]. The cell surface expression of CD63 is mediated by tumor-associated antigen L6, L6-Ag [10]. CD63 is abundantly expressed as a surface antigen in the early stage of melanoma, but its expression decreases with malignant progression [11], suggesting a negative correlation between CD63 surface levels and invasiveness.

TM4SF5 is related to the tetraspanins by having four transmembrane domains, but belong to transmembrane 4 L6 family member 5 due to no CCG motifs in the second extracellular loop [12], [13]. Being similar to tetraspanins, TM4SF5 has an intracellular loop, two extracellular loops, and cytosolic NH_2_- and COOH-terminal tails [12], [13]. TM4SF5 is induced by TGFβ1/Smads signaling pathway in fibrotic livers of CCl_4_-administarated mice [14]. More than 80% of HCC is known to be associated with advanced fibrosis or cirrhosis [15], [16]. TM4SF5 is highly expressed in hepatocellular cancer tissues, and enhances their aberrant proliferation, migration, and invasion of hepatocytes [13]. TM4SF5 mediates adhesion-dependent focal adhesion kinase (FAK)/c-Src activation to direct motility and invasive capacity [17], [18]. Although TM4SF5 does not belong to the genuine tetraspanin family [12], TM4SF5 can form TEMs with other tetraspanins and can play a role in the regulation of metastasis. Furthermore, any hierarchy among these tetraspan(in)s has not been reported.

Here in this study, we examined the correlations between TM4SF5, CD151, and CD63 expression using normal Chang hepatocytes that do not express TM4SF5, chronically TGFβ1-treated Chang cells that do express TM4SF5 [14], and other hepatocyte cells. We found that TM4SF5 expression could override CD151 functions, and TM4SF5 acted antagonistically to CD63 during liver fibrosis development and during hepatic migration/invasive extracellular matrix (ECM) -degradation.

## Materials and Methods

### Cell Culture

Normal human hepatocyte Chang cells and chronically TGFβ1-treated Chang cells (Chang-TGFβ1), hepatocellular carcinoma Huh7, Hep3B, SNU449, non-small cell lung cancer (NSCLC) HCC827 cells were described previously [19]. Chang, SNU449, and HCC827 cells do not express TM4SF5, whereas Chang-TGFβ1, Huh7, and Hep3B cells express TM4SF5 [19]. Cells including stable Huh7-shScramble (TM4SF5-expressing) or Huh7-shTM4SF5 (TM4SF5-suppressed) cells were maintained in RPMI-1640 (WelGene, Daegu, Korea) containing 10% FBS and antibiotics (Invitrogen, Grand Island, NY, USA).

### Extract Preparation and Western Blots

Subconfluent cells in media containing 10% FBS, or cells transiently transfected with short hairpin RNA (shRNA, control shRNA or shRNA against TM4SF5, CD151, or CD63) separately or in combination with each shRNA or cDNA plasmid encoding for FLAG-TM4SF5, Strep-TM4SF5, CD151, or CD63, for 48 h were harvested for whole cell lysates using radio-immunoprecipitation assay (RIPA) lysis buffer containing 0.1% SDS, 0.5% deoxycholate, 1% NP-40, and proteinase inhibitors [19]. Tissue extracts from human or mouse livers were also prepared as previously reported [19]. The primary antibodies included anti-α-tubulin (Sigma, St Louis, MO, USA), anti-CD151, anti-CD63, anti-pY^416^c-Src, anti-FLAG (Cell Signaling Technol. Danvers, MA, USA), anti-FAK (BD Transduct. Lab., Bedford, MA, USA), anti-pY^397^FAK (Abcam, Cambridge, UK), anti-c-Src, anti-pY^577^FAK (Santa Cruz Biotech., Santa Cruz, CA, USA), and anti-TM4SF5 [19].

### Coimmunoprecipitations

Whole cell extracts for coimmunoprecipitation were prepared by using a lysis buffer (10 mM Tris, pH 7.5, 150 mM NaCl, 1 mM CaCl_2_, 1 mM MgCl_2_) including 1% Brij97 and protease inhibitors. The whole cell lysates were then immunoprecipitated with anti-FLAG antibody-precoated agarose beads (Sigma) overnight. Alternatively, whole cell extracts from Chang-TGFβ1 cells, or extracts from Chang or HCC827 cells transiently transfected with Strep-tagged mock or TM4SF5 were incubated with either normal immunoglobulin (IgG), anti-CD151 antibody (Cell Signaling Technol.), or biotin-precoated beads (IBA, Hanover, Germany) for 2 h. Immunoprecipitated proteins were boiled in 2× SDS-PAGE sample buffer before Western blot analysis.

### Flow Cytometry

Cells were analyzed by flow cytometry for tetraspanin expression profiles, as previously reported [20]. Primary antibodies used included anti-CD151, anti-CD63, anti-CD9, and anti-TM4SF5 (Clone # 27, anti-TM4SF5 mAb, described below).

### TM4SF5 Antibody

Generation of anti-TM4SF5 monoclonal antibodies were described previously [21]. Briefly, a recombinant TM4SF5 LEL domain (amino acid residues 113 to 157) fused with Fc (i.e., an antibody ***fragment*** crystallizable) and myc at the C-terminus was expressed in HEK293E cells, purified by affinity chromatography using protein A/G-agarose (Millipore, Billerica, MA, USA), and used as an antigen to select antibodies. An anti-TM4SF5 monoclonal antibody (human Fc-fusion form) was purified by affinity chromatography using protein A/G-agarose and used in flow cytometry or immunohistochemistry analysis.

### Indirect Immunofluorescence

Cells grown on glass coverslips without or with transient transfection with FLAG-TM4SF5 or CD151 for 48 h or cells on coverslips were immunostained using antibody against CD151, CD63, TM4SF5, and/or FLAG, in addition to DAPI staining for nucleus. In cases, LAMP2, a lysosomal marker, was immunostained using anti-LAMP2 (Abcam, Cambridge, UK). Immunofluorescent images were acquired on a fluorescent microscope (BX51TR, Tokyo, Olympus) or a confocal laser scanning microscope (Nikon C2, Nikon, Tokyo, Japan).

### Transwell Migration Assay

Cells transfected with diverse shRNA or plasmids were processed for the Transwell migration assay using 8-µm pore transwells (Corning, Corning, NY, USA), as described previously [22]. The migration assay was performed for 18 h with normal 10% FBS-containing media in the lower chambers. Migrating cells were stained and visualized using microscopy, and representative images were obtained. Mean values ± standard deviation were evaluated from randomly saved images and were graphed.

### ECM-Degradation Analysis

ECM-degradation by cells on coverslips precoated with Oregon Green 488-conjugated-gelatin (Invitrogen) was analyzed for 18 h, as described in a previous report [22].

### TM4SF5 promoter assay

The transcriptional activity of the TM4SF5 promoter (−3.2∼+0.5 kb fragment in pGL3) was analyzed, as previously described [14]. The total DNA amount for each transfection was normalized using control DNA.

### Immunohistochemistry of Murine Fibrotic or Human Tumor Liver Tissues

All animal procedures were performed in accordance with the procedures of the Seoul National University Laboratory Animal Maintenance Manual and Institutional Review Board (IRB) agreement approved by Institute of Laboratory Animal Resources Seoul National University (ILARSNU). Four-week-old mice (BALB/c, Orient. Co. Ltd, Seungnam, Korea) were housed in a specific pathogen-free room with controlled temperature and humidity. Mice aged 5 weeks (n = 5) were injected intraperitoneally with or without CCl_4_ (Sigma, 1 mg/kg) in 40% olive oil, three times per week for 2 weeks. Double-immunostaining of human liver tissues were obtained from each patient as reported previously after informed content [19], or murine liver tissues were incubated with primary antibodies specific for TM4SF5 [21] (Clone # 27, anti-TM4SF5 mAb, see above), CD63, and CD151 (Cell Signaling Technol.) with or without DAPI staining. Alternatively, the tissues were processed for Masson’s trichrome or hematoxylin and eosin staining, as previously described [23].

### Statistical Methods

Student’s *t*-tests were performed for statistical comparisons of mean values to determine significance. A *P* value less than 0.05 was considered statistically significant.

### Supplementary Information

Supplementary figures can be found at the journal web site in File S1.

## Results

### Correlations between TM4SF5, CD151, and CD63 Expression Levels

**Figure 1:**
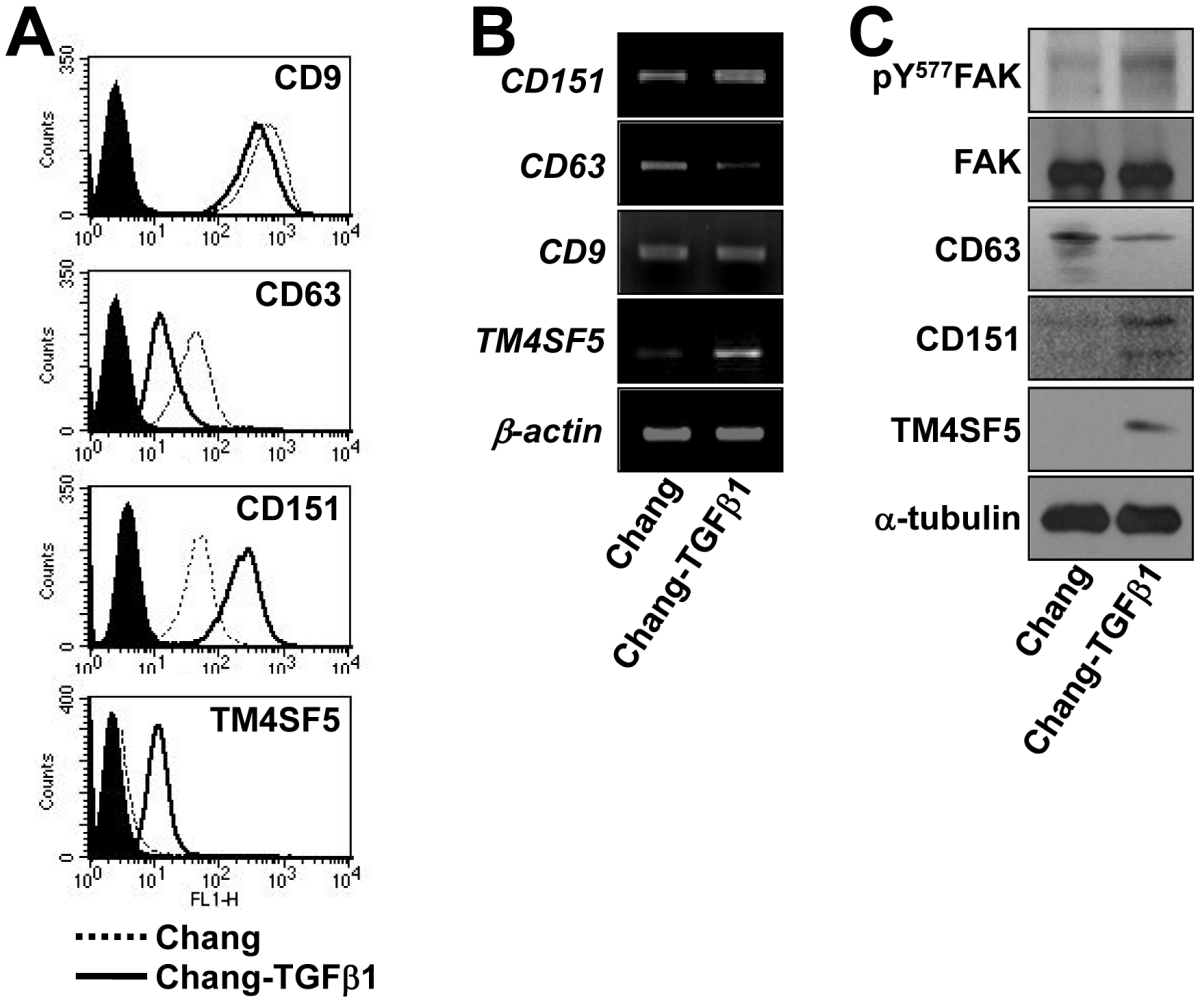
Differential relationships of TM4SF5, CD151, and CD63 expression. (A to C) TM4SF5-null Chang (normal hepatocyte) and TM4SF5-expressing Chang-TGFβ1 (Chang cells chronically treated with TGFβ1) cells were analyzed for CD9, CD63, CD151, and TM4SF5 expression by flow cytometry (A), by RT-PCR (B), and by Western blot (C). Data represent three independent experiments.

To determine the inter-relationships between the tetraspanins and TM4SF5, we examined the expression profiles of certain tetraspanins (i.e., CD9, CD63, CD82, CD105, CD117, and CD151) and TM4SF5 using normal human liver hepatocyte Chang cells and Chang-TGFβ1 cells that were derived by chronic culturing in TGFβ1-containing media, leading to TM4SF5 expression [14]. Flow cytometry analyses showed that Chang-TGFβ1 cells expressed TM4SF5, whereas the control parental Chang cells did not; additionally, the Chang-TGFβ1 cells expressed more CD151 but less CD63 on the cell surface. However, levels of CD9, CD82, CD105, and CD117 did not change (Fig. 1A and data not shown). Therefore, a positive correlation was found between TM4SF5 and CD151, and a negative correlation was found between TM4SF5 and CD63 at both the mRNA (Fig. 1B) and protein levels (Fig. 1C). In addition to increased TM4SF5 expression, Chang-TGFβ1 cells also showed increased FAK activity (i.e., pY^577^FAK). Since the intracellular loop (ICL) of TM4SF5 binds to and activates FAK to direct persistent migration [17].

### The Relationships between TM4SF5, CD151, and CD63 were Maintained during the Development of Liver Malignancies

**Figure 2:**
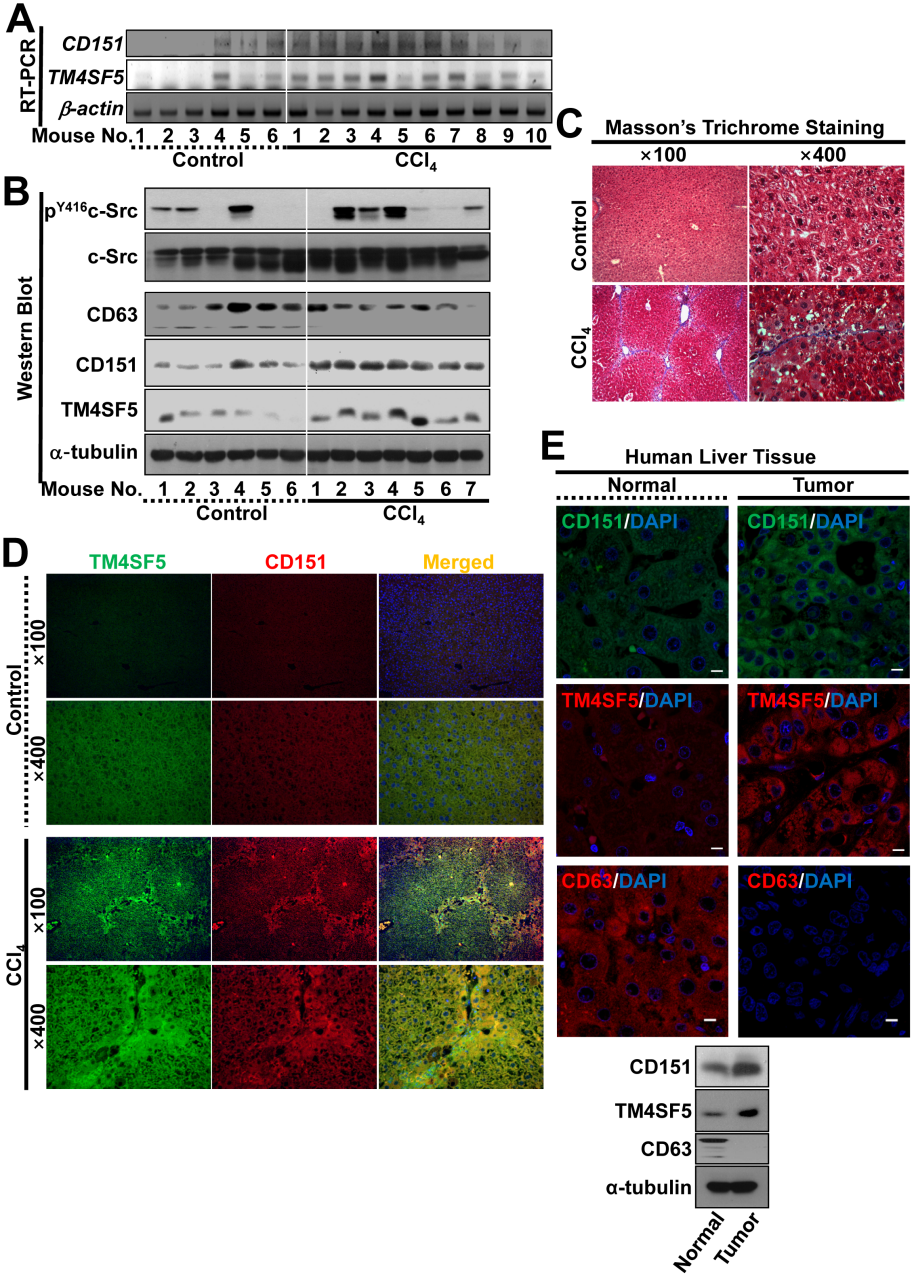
The relationships of TM4SF5, CD151, and CD63 in the development of murine liver fibrosis and liver cancer. (A to D) Liver tissues from mice administrated with control vehicle or CCl_4_ every other day for 2 weeks were used for RT-PCR analysis (A), harvested for whole extracts, prior to Western blots for the indicated proteins (B), used for Masson’s trichrome staining to determine collagen I expression (C), or processed for immunohistochemistry with double-staining for TM4SF5 and CD151 (D). (E) Human normal or liver tumor tissues were processed for Western blots or immunohistochemistry to identify CD151 (top), TM4SF5 (middle), and CD63 (bottom) and the nuclei were stained using DAPI. Scale bars depict 10 µm. Data represent three independent experiments.

We examined the relationships of the tetraspanins in control mouse livers and CCl_4_-treated mouse fibrotic livers. A positive correlation between *TM4SF5* and *CD151* mRNA levels was more obvious in the CCl_4_-treated mouse livers, than in the control livers (Fig. 2A). A positive relationship between CD151 and TM4SF5 and a concomitant negative relationship between CD63 and TM4SF5 were observed in the CCl_4_-administered mouse livers but not in the control livers (Fig. 2B). Further, whole liver extracts prepared from CCl_4_-treated mice showed generally higher c-Src activities, compared with those from control mice (Fig. 2B), as expected from the previous study showing that c-Src activity is downstream of TM4SF5 for cellular invasion [18]. In the CCl_4_-treated mouse livers, collagen I was deposited along the fibrotic septa, as observed after Masson’s Trichrome staining (Fig. 2C), and the co-localization of CD151 and TM4SF5 was observed after immunofluorescent double-staining (Fig. 2D). We next examined the relationship between CD151, CD63, and TM4SF5 in human liver cancer tissues. Liver tumor tissues with high levels of TM4SF5 expression also showed higher levels of CD151 expression, but very lower levels of CD63, compared with those in normal liver tissues (Fig. 2E). These observations demonstrated that a positive relationship existed between TM4SF5 and CD151 and that a negative relationship existed between CD63 and TM4SF5; this correlationship in expression could be involved in liver malignancy.

### Different Hepatocytes Exhibited Differential Relationships between TM4SF5, CD151, and CD63 Expression at the Transcriptional Level

**Figure 3:**
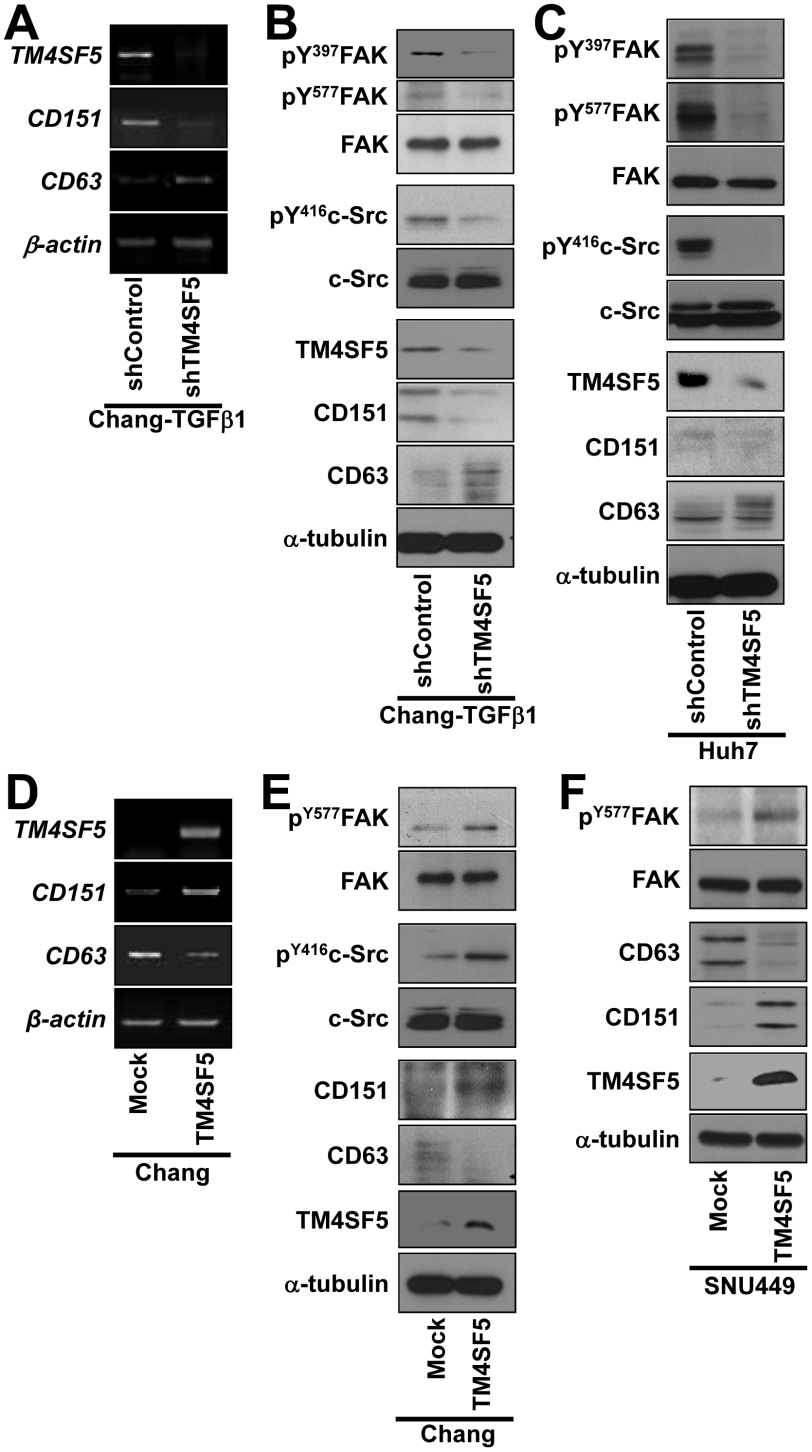
TM4SF5 expression positively or negatively regulated CD151 and CD63 expression levels, respectively, and altered migratory signaling activity. (A to C) TM4SF5-expressing Chang-TGFβ1 (A and B) or Huh7 cells (C) transfected with shRNA against TM4SF5 (shTM4SF5) or a control-scrambled sequence (shControl) were processed for RT-PCR (A) or Western blots (B and C) against the indicated molecules. (D to F) TM4SF5-null Chang (D and E) or SNU449 (F) cells transfected with TM4SF5 cDNA or control plasmids (Mock) were processed for RT-PCR (D) or Western Blots (E and F). Note that CD63 immunoblots showed multiple bands, presumably due to 3 possible isoforms with *N-*glycosylations (http://www.uniprot.org/uniprot/P08962). Data represent three different experiments.

We also examined whether CD151 and CD63 levels were altered when TM4SF5 expression was suppressed in Chang-TGFβ1 cells and Huh7 cells that endogenously express TM4SF5. In contrast to the control shRNA transfected cells, Chang-TGFβ1 cells transfected with shTM4SF5 had decreased levels of CD151 and concomitantly increased levels of CD63 mRNA and proteins (Figs. 3A and B). These relationships were also observed in Huh7 cells (Fig. 3C). Additionally, FAK and c-Src phosphorylation in the Chang-TGFβ1 and Huh7 cells decreased upon TM4SF5 suppression (Figs. 3B and C). TM4SF5 interacts with and activates FAK and c-Src, resulting in enhanced migration and invasion [17], [18]. When TM4SF5 was ectopically expressed in Chang or SNU449 cells, CD151 mRNA and protein levels increased along with FAK and c-Src activities, whereas CD63 levels decreased (Figs. 3D, E, and F).

**Figure 4:**
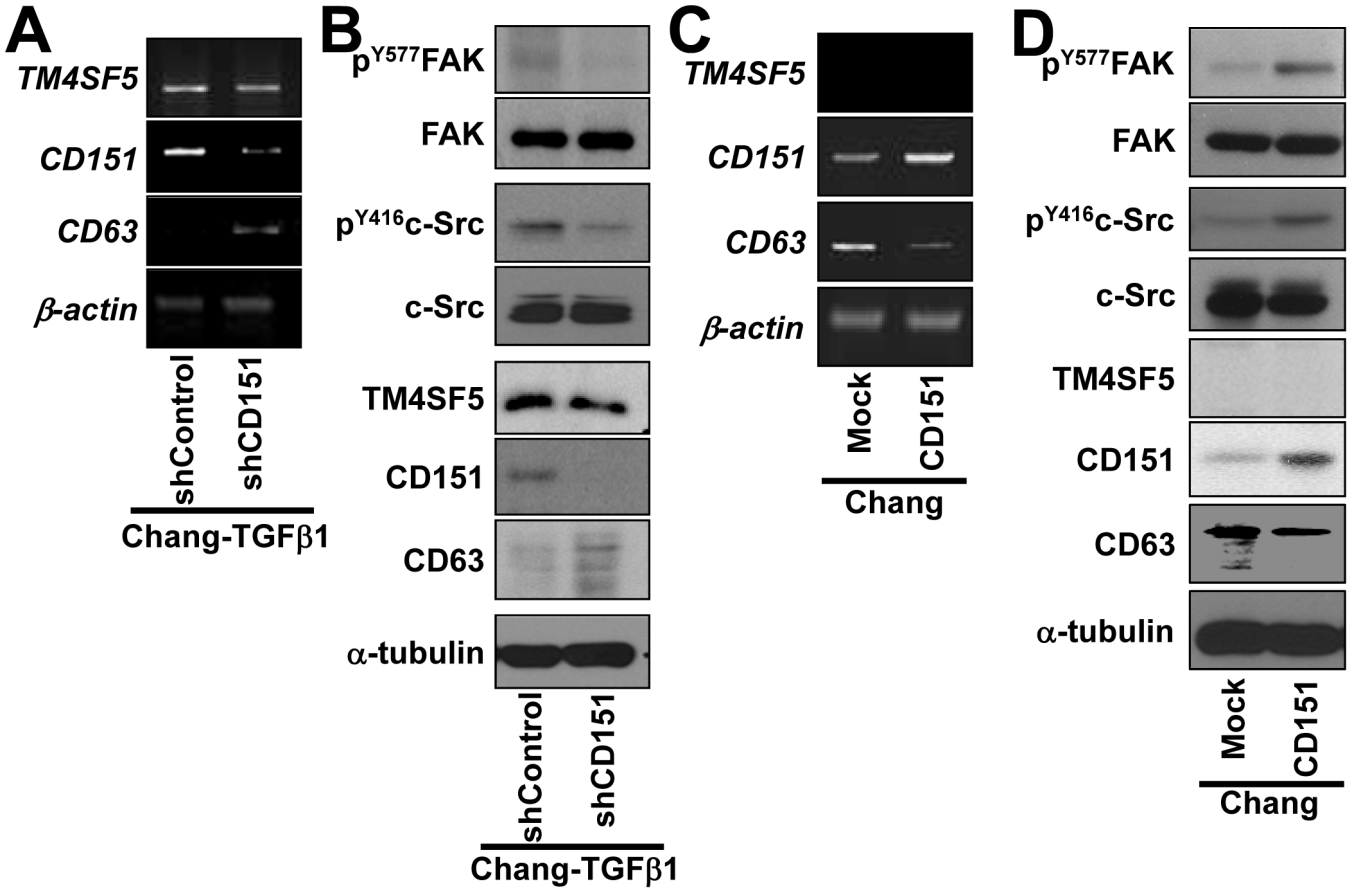
CD151 regulated CD63 expression, but not TM4SF5 expression. Chang-TGFβ1 cells were transiently transfected with shRNA against CD151 (shCD151) or a control-scrambled sequence (shControl) for 48 h (A and B). Chang cells were transiently transfected with CD151 cDNA or a control plasmid (Mock) for 48 h (C and D). The cells were then processed for RT-PCR (A and C) or for standard Western blots (B and D) for the indicated molecules. Data represent three different experiments.

We next examined whether the modulation of CD151 expression might affect the expression of TM4SF5 or CD63. CD151 suppression in Chang-TGFβ1 cells did not change the mRNA or protein levels of TM4SF5, but suppression did increase CD63 expression levels (Figs. 4A and B). However, c-Src and FAK activities were slightly reduced, presumably because CD151 is also important for their activation, as shown in melanoma cells [24]. Overexpression of CD151 in Chang cells decreased the levels of CD63 mRNA and protein, although the endogenous (usually null) levels of TM4SF5 mRNA and protein were not altered (Figs. 4C and D). The lack of change in TM4SF5 expression after alteration of CD151 expression supports the hypothesis that TM4SF5 is upstream of CD151.

**Figure 5:**
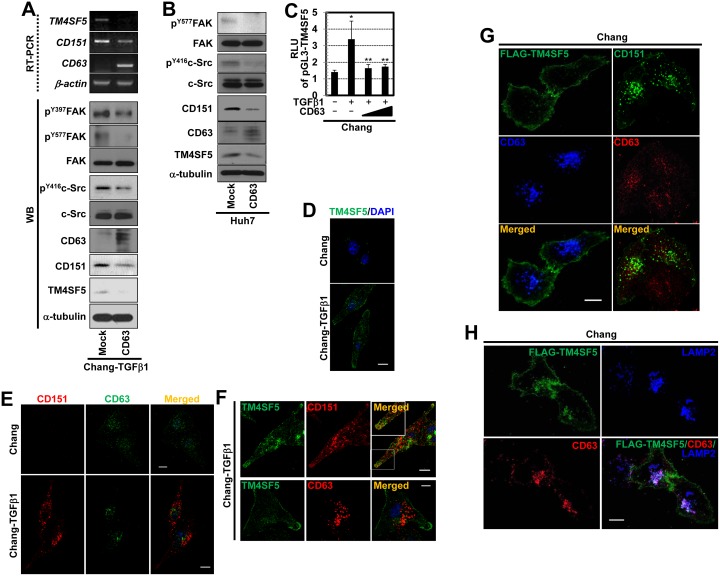
CD63 was antagonistic to TM4SF5 and CD151. (A and B) Chang-TGFβ1 cells (A) or Huh7 cells (B) transfected with CD63 cDNA or a control plasmid (Mock) were processed for RT-PCR or Western blots. (C) Chang cells transfected with pGL3-TM4SF5 promoter DNA without or with CD63 cDNA for 24 h were treated with vehicle or TGFβ1 for additional 24 h, prior to luciferase reporter gene assay. Each value was shown at mean ± standard deviation. * depicts statistical significance (*p*<0.05) and ** depicts insignificance (*p*≥0.05). (D and E) Chang and Chang-TGFβ1 cells were immunostained for TM4SF5 (green, D), CD63 (green, E), or CD151 (red, E) in addition to nuclear staining using DAPI. (F) Chang-TGFβ1 cells were immunostained for TM4SF5 (green) and either CD151 (top panel) or CD63 (bottom panel) in addition to DAPI staining. White box depicts area for an enlarged insert. (G) Chang cells transiently transfected with FLAG-TM4SF5 or CD151 were immunostained for CD63 (blue or red) and either FLAG-TM4SF5 (green) or CD151 (green) prior to visualization by confocal microscopy. (H) Chang cells transfected with FLAG-TM4SF5 for 48 h were immunostained using anti-FLAG (green), anti-LAMP2 (a lysosomal marker, blue), and anti-CD63 (red) antibody, prior to visualizations using confocal microscopy. Scale bars depict 10 µm. Data represent three independent experiments.

We further examined if the modulation of CD63 levels affected TM4SF5 or CD151 levels. Overexpression of CD63 in Chang-TGFβ1 cells decreased TM4SF5 and CD151 mRNA and protein levels and consequently decreased c-Src activity; however, FAK activity was unchanged (Fig. 5A). This relationship was also observed in Huh7 cells transfected with CD63 (Fig. 5B). Further, TGFβ1 treatment resulted in an enhanced transcriptional activity of TM4SF5 promoter (Fig. 5C), which was declined to the basal level by CD63 introduction (Fig. 5C), indicating an antagonistic effect of CD63 on TM4SF5 transcription.

### TM4SF5 and CD151 Interact at the Membrane Surface, and TM4SF5 Mediates the Exclusion of CD63 from the Membrane Surface

We next examined the expression of TM4SF5 in Chang or Chang-TGFβ1 cells via immunofluorescence microscopy. Whereas TM4SF5 was very minimally detected in Chang cells, it was obviously shown on the plasma membranes and in cytosolic compartments of Chang-TGFβ1 cells (Fig. 5D). Further, when CD63 and CD151 were double immunostained, Chang cells showed CD63 both on plasma membrane and in cytosol but showed a hardly detectable expression level of CD151 (Fig. 5E, upper panels). However, Chang-TGFβ1 cells showed CD151 throughout a cell and CD63 mostly in cytosolic area, without any co-localization between CD151 and CD63 in cytosol (Fig. 5E, lower panels). In addition, when endogenous TM4SF5 and either CD151 or CD63 in Chang-TGFβ1 cells were immunostained, TM4SF5 were partially co-localized with CD151 around protrusive tips but not with CD63 that was mostly in cytosol compartments (Fig. 5F).

We then examined whether the localization of CD63 might be influenced by TM4SF5. Immunostaining of CD63 in Chang cells transfected with FLAG-TM4SF5 revealed the translocation of CD63 to endosomal regions around the nucleus (Fig. 5G, left panels), whereas the effects of transfection with CD151 on CD63 localization were obviously insignificant since both CD151-transfected and -untransfected (neighboring) cells showed a similar pattern in CD63 localization (Fig. 5G, right panels). Therefore, cytosolic localization of CD63 in Chang-TGFβ1 cells (Fig. 5E, lower panel) could be due to TM4SF5 overexpression in those cells. Interestingly, when TM4SF5 was exogenously expressed in Chang cells, CD63 was mostly co-localized with LAMP2, a lysosome marker (Fig. 5H). These observations indicate that TM4SF5 increased CD151 expression levels (Figs. 3D and E) leading to enhanced tumorigenic roles of CD151, and at the same time caused the translocation of CD63 from the membrane surface to the lysosomal membranes, possibly leading to the inhibition of the tumor-suppressive roles of CD63.

**Figure 6:**
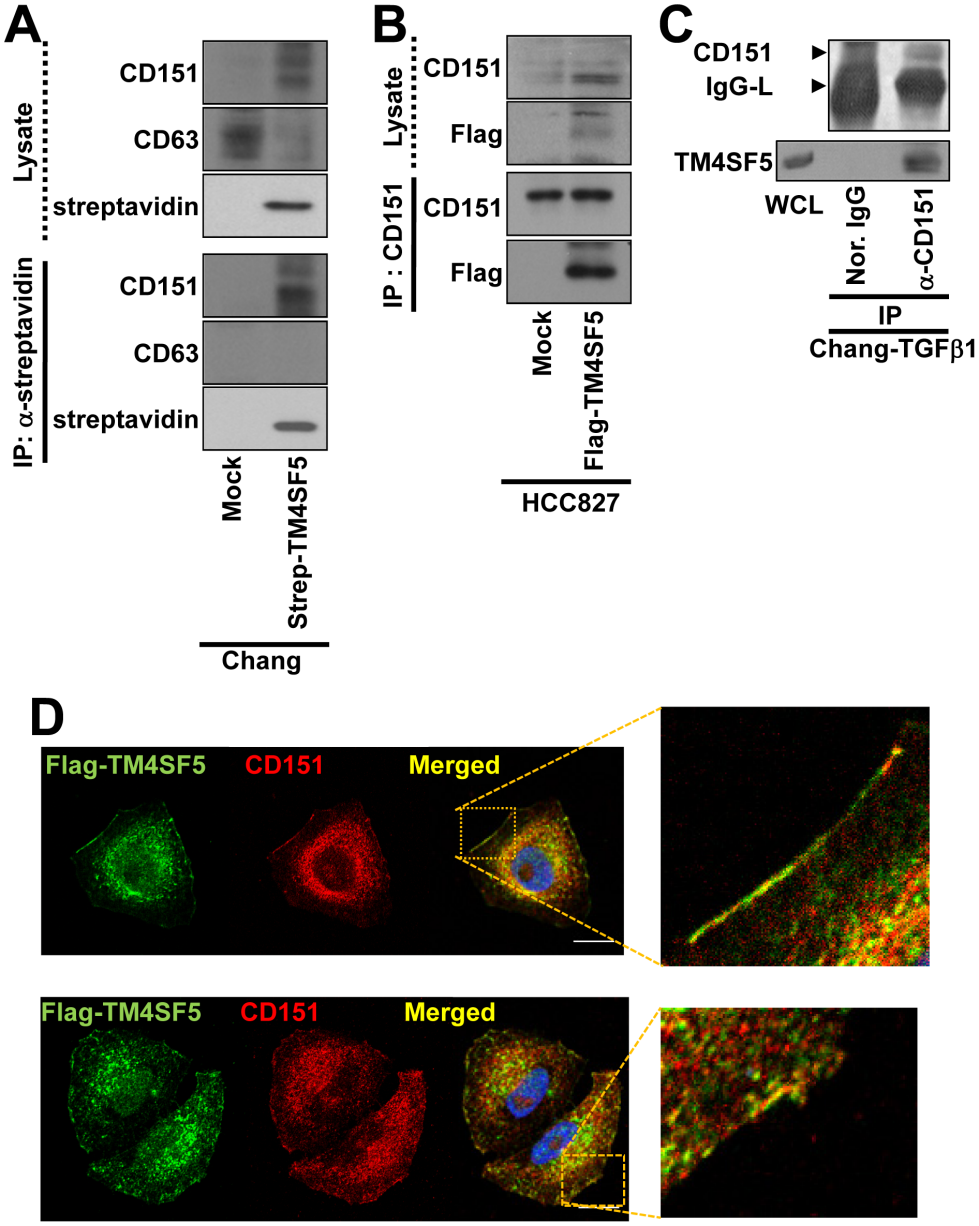
TM4SF5 coimmunoprecipitated with CD151. (A) Whole cell lysates from Chang cells transfected with control Strep or Strep-TM4SF5 plasmid were immunoprecipitated (IP) with anti-streptavidin-coated agarose beads before immunoblotting using anti-CD151, anti-CD63, or anti-streptavidin. (B) HCC827 lung carcinoma cells transfected with FLAG-mock or FLAG-TM4SF5 were harvested for whole cell extracts. The extracts were immunoprecipitated (IP) with anti-CD151 antibody prior to immunoblotting for FLAG and CD151, in parallel with lysates. (C) Whole cells extracts from Chang-TGFβ1 cells were immunoprecipitated with normal (Nor.) IgG or anti-CD151 (α-CD151) antibody, prior to standard Western blots for CD151 or TM4SF5, in parallel with whole cell lysates (WCL). (D) Chang cells transfected with FLAG-TM4SF5 were immunostained for CD151 (red) and FLAG (green), prior to visualization using a confocal microscope. Note that some TM4SF5 co-localized with CD151 at the membrane surface and internally in endosomal regions, but other TM4SF5 localized independently of CD151 even on the membrane surface. The images were presented in an independent duplicate for the same condition. Data represent three independent experiments.

We next examined the relationship between TM4SF5 and CD151 at the membrane surface. CD151 coimmunoprecipitated strep-tagged TM4SF5 in Chang cells, whereas CD63 did not (Fig. 6A); neither protein coimmunoprecipitated with TM4SF5 in the mock reaction, indicating a possible physical interaction between TM4SF5 and CD151. This interaction was also observed in HCC827 non-small lung cancer cells transfected with FLAG-TM4SF5 but not with mock plasmids (Fig. 6B). In addition, endogenous TM4SF5 in Chang-TGFβ1 cells was shown to be co-immunoprecipitated with CD151 (Fig. 6C). Furthermore, confocal immunofluorescent images indicated that TM4SF5 and CD151 co-localized at the membrane boundaries. However, this co-localization was not always observed, so that either TM4SF5 or CD151 alone could also localize to the membrane boundaries and intracellular areas near the membrane surfaces (Fig. 6D).

### Cross-talks between TM4SF5, CD151, and CD63 Regulates Cell Migration and Invasive ECM Degradation

**Figure 7:**
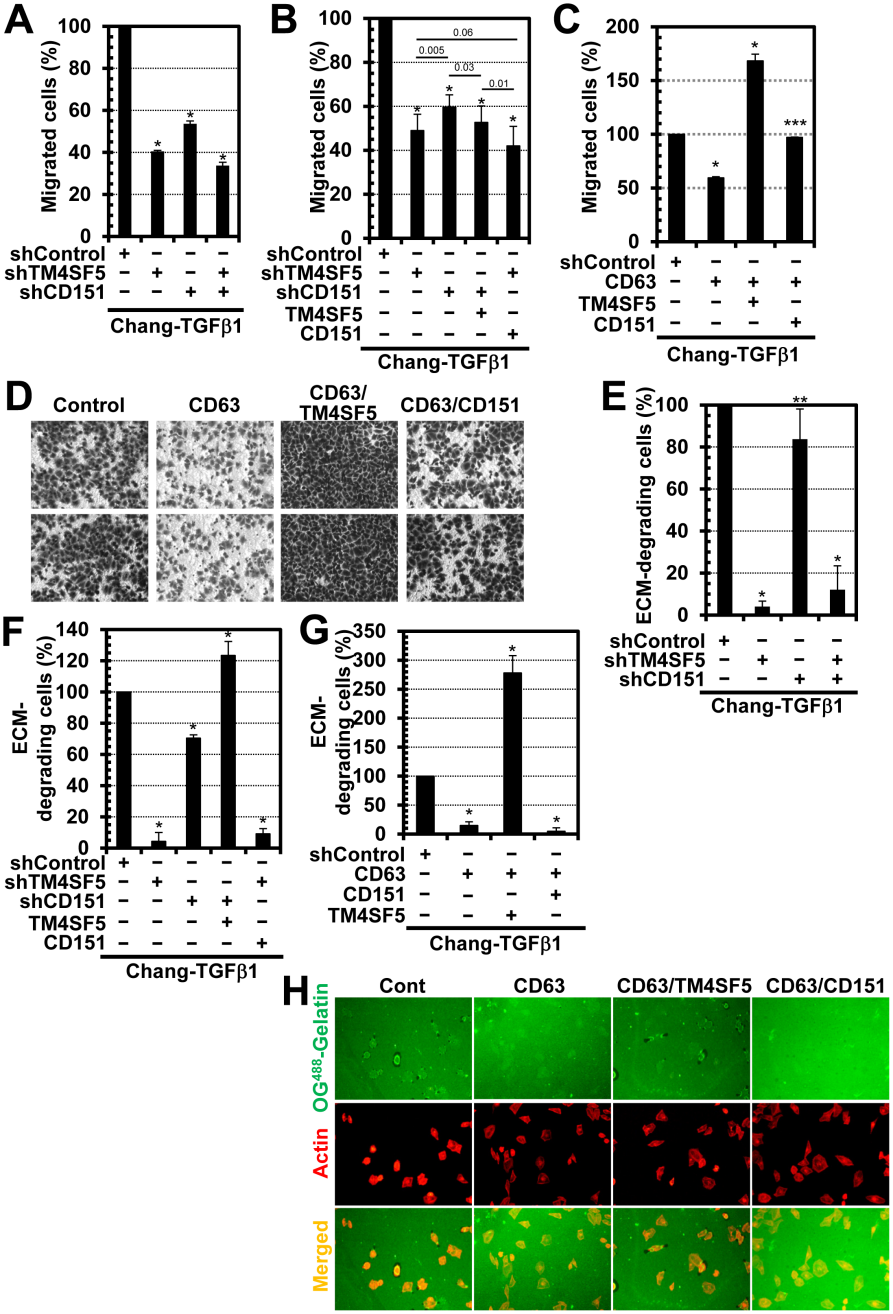
Different collaborative effects of TM4SF5, CD151, and CD63 on migration and invasive ECM degradation. Transwell migration analyses (A to D) or ECM-degradation analyses (E to H) were performed using Chang-TGFβ1 cells transfected with the indicated shRNAs or plasmids. (A to D) The bottom chamber was filled with 10% FBS/DMEM-H. After 18 h, cells migrated to the bottom surface of the filter were stained and imaged. Representative images (at least 5 images) were counted to determine migration in each experimental condition. Mean ± standard deviation were graphed (A to C), and representative images of (C) were shown (D). (E to H) Chang-TGFβ1 cells transfected with shRNA or plasmids were reseeded onto coverslips precoated with Oregon Green 488-conjugated gelatin and incubated for 18 h in a CO_2_ incubator. The dark-spotted ECM-degraded areas from more than 5 random images were counted for graphic presentations using mean ± standard deviation. Representative images in (H) are shown (G). * or ** depict *P* values less than 0.001 or 0.05 for statistical significance, respectively, whereas *** depicts *P* values greater than 0.05 for insignificance, and numbers in (B) represent the *P* values by Student’s *t*-tests. Data represent three independent experiments.

We next investigated the biological functions of these relationships in the regulation of migration and invasive ECM-degradation. The cells were first analyzed using a Transwell migration assay in which normal culture media including 10% serum was added to the lower chamber. When Chang-TGFβ1 cells were transfected with shRNA against TM4SF5 (shTM4SF5) or CD151 (shCD151), either suppression of TM4SF5 or CD151 alone or in combination significantly decreased cellular migration; however, TM4SF5 suppression alone decreased the cells’ migration capacity more than CD151 suppression alone (Fig. 7A and Fig. S1 in File S1). The decreased migration, caused by either TM4SF5 or CD151 suppression, did not recover after the addition of CD151 or TM4SF5, but was instead slightly-reduced (Fig. 7B and Fig. S2 in File S1). This observation suggests that there might be specific relationships between the TM4SF5- and CD151-mediated migrations, by which the migration could become minimal by suppression of either of both molecules. Overexpression of CD63 in Chang-TGFβ1 cells decreased the migratory capacity of these cells, but the addition of CD151 allowed cells to recover to their basal migratory level. On top of the CD63 overexpression, the addition of TM4SF5 increased the cells’ migratory capacity above the basal levels (Figs. 7C and D). This result indicates that the effects of TM4SF5 were stronger, compared to CD63 and CD151, and both CD151 and TM4SF5 antagonized CD63 during migration.

Next, the effect of each molecule on invasive ECM degradation was analyzed. Chang cells were not able to degrade the ECM under our experimental conditions (data not shown), whereas Chang-TGFβ1 cells could (Figs. 7E and S3 in File S1). TM4SF5 suppression in Chang-TGFβ1 cells almost completely abolished the ECM degradation capacity, whereas CD151 suppression only slightly inhibited ECM-degradation, compared to the shControl-transfected cells (Figs. 7E and S3 in File S1). Furthermore, the suppression of both TM4SF5 and CD151 significantly inhibited ECM-degradation (Figs. 7E and S3 in File S1), suggesting a dominant effect of TM4SF5 over CD151 in ECM degradation. When TM4SF5 was expressed in CD151-depleted cells, ECM-degradation was enhanced and occurred at a level higher than the level in shControl-transfected cells (Figs. 7F and S4 in File S1). However, the expression of CD151 in TM4SF5-depleted cells did not change the level of ECM degradation, which remained almost completely inhibited (Figs. 7F and S4 in File S1). CD63 overexpression in Chang-TGFβ1 cells completely abolished ECM-degradation, which was recovered to a rather enhanced level by the addition of TM4SF5, but not by the addition of CD151 (Figs. 7G and H). This observation indicates that TM4SF5 plays major roles in invasive ECM degradation, and can overwhelm the effects caused by CD151 and CD63.

## Discussion

This study revealed that TM4SF5 levels were positively correlated with CD151 expression but were negatively with CD63 expression during liver fibrosis and tumorigenesis. TM4SF5 collaborated with CD151 for migration but played a more important role than CD151 in invasive ECM-degradation. TM4SF5 appeared to bind CD151 on the membrane surface for roles in cell migration. However, CD63 was excluded from the membrane surface, where it plays a tumor-suppressive role, by the expression of TM4SF5 and/or CD151. Therefore, it is likely that TM4SF5 may collaborate with CD151 to regulate cell migration, but TM4SF5 overrides CD151 during invasion by promoting the internalization of CD63 from the membrane surface to the lysosomes. This action may decrease the tumor-suppressive functions by CD63, during TM4SF5-mediated liver malignancies.

Tetraspanins are located in TEMs and are involved in cellular adhesion, migration, and invasion via homophilic and/or heterophilic interactions among the tetraspanins, integrins, and growth factor receptors [25]. TM4SF5 interacts with integrins α2β1 [26] and α5 [27], and with EGFR [28]. CD151 interacts with laminin-binding integrins tetraspanins [4], and CD63 also interacts with integrins and tetraspanins [9]. The present study revealed an interaction between TM4SF5 and CD151, however this interaction might further be associated to other membrane proteins, including tetraspanins, integrins α2β1 and/or α5β1, and growth factor receptors. In a putative TM4SF5-enriched microdomain (i.e., T_5_EM) containing TM4SF5, interactions among the membrane proteins including CD151 could transduce intracellular signaling and regulate cellular functions. A mechanistic evaluation of the composition of the T_5_EMs and their roles in directional migration is currently underway, although TM4SF5 is not a member of but rather related to tetraspanins.

Although TM4SF5, CD151, and CD63 can localize to the membrane surface and within the endosome system, their localizations could play critical roles in tumor progression and fibrotic phenotype development in the livers. Fibrosis and tumorigenesis commonly involve cell migration and epithelial-mesenchymal transition (EMT) processes [29]. TM4SF5 regulates RhoA/Rac1 activity during EMT [19], binds through its intracellular loop and activates FAK for a directional migration [17], and binds through its cytosolic C-terminus to facilitate c-Src activation and invasion [18]. CD151 expression also causes FAK activation [24], and engagement of laminin-binding integrins in CD151-expressing cells to activate the RhoA GTPase family during cell motility [30]. We found that a certain population of TM4SF5 protein on the membrane surface is bound to CD151. Thus, at the membrane edges TM4SF5 and CD151 might synergistically function in cell migration. However, the collaborations between TM4SF5 and CD151 appeared limited. Some amount of each protein was separately localized on the membrane boundary, and cell migration inhibited by either TM4SF5 or CD151 suppression was not restored by the addition of CD151 or TM4SF5, respectively. Thus, TM4SF5 or CD151 alone might be separately involved in cell migration, as previously reported [17], [30].

CD63 is localized either on the plasma membrane or on intracellular vesicles co-localized with the markers for late lysosomal compartments, and CD63 localization at the membrane surface increases by suppression of TM4SF1 [10]. CD63 at the membrane surface associates with TM4SF1 (L6-Ag) to enact its cell migration effects [10]. TM4SF1 is another member of transmembrane 4 L6 family and shares approximately 50% sequence identity with TM4SF5 [12]. Similar to TM4SF1, TM4SF5 expression resulted in the internalization of CD63 from the cell surface to the lysosomes, thus decreasing CD63 level on the membrane surface and reducing its tumor suppressive actions. Thus, it is likely that the regulation of one T_(5)_EM component controls other components of the T_(5)_EM, leading to the regulation of cell motility.

We observed that cell migration was significantly blocked by CD63 expression. However, the addition of TM4SF5 into CD63-transfected cells resulted in enhanced migration at levels higher than observed in the controls, in addition to the recovery of CD63-inhibited migration. The addition of CD151 into CD63-transfected cells only resulted in recovery of the CD63-induced inhibition of migration. CD63 expression completely decreased ECM-degradation; the ECM degradation was dramatically enhanced by the addition of TM4SF5. However, this inhibition was not overcome by the overexpression of CD151. Furthermore, CD151 suppression slightly inhibited ECM-degradation, compared with the stronger inhibition caused by TM4SF5 suppression. Overexpression of TM4SF5 greatly enhanced ECM degradation beyond the basal levels, in addition to its capacity to overcome the inhibition of ECM degradation caused by CD151 suppression- or CD63 overexpression. Interestingly, the CD63-mediated inhibition of migration or ECM-degradation was recovered or unaltered by CD151 overexpression, respectively, suggesting that CD151’s effects were stronger than CD63’s effects on migration but had no effect on invasive ECM-degradation.

The current study suggests a hierarchical relationship among the tetraspanins of TM4SF5-containing TEMs (i.e., T_5_EMs), and suggests that these TEMs are involved in cell migration and invasion. Therefore, the components of these TEMs may be promising target(s) for future treatments of liver malignancy.

## Supporting Information

File S1 **Contains the files: Figure S1.** Transwell migration analysis was performed for Chang-TGFβ1 cells transfected with the indicated shRNAs or plasmids. The bottom chamber was filled with 10% FBS/DMEM-H and cells were loaded to upper chamber. After 18 h, cells migrated to the bottom surface of the filter was stained and imaged. Representative images more than at least 5 images were considered for counting of migrated cells in each experimental condition. Images of a representative experimental set were shown for each experimental condition. Data represent three independent experiments. **Figure S2.** Transwell migration analysis was performed for Chang-TGFβ1 cells transfected with the indicated shRNAs or plasmids. The bottom chamber was filled with 10% FBS/DMEM-H and cells were loaded to upper chamber. After 18 h, cells migrated to the bottom surface of the filter was stained and imaged. Representative images more than at least 5 images were considered for counting of migrated cells in each experimental condition. Images of a representative experimental set were shown for each experimental condition. Data represent three independent experiments. **Figure S3.** Chang-TGFβ1 cells transfected with shRNA against TM4SF5 (shTM4SF5) or CD151 (shCD151) were reseeded on coverglasses precoated with Oregon Green® 488-conjugated gelatin and incubated for 18 h in a CO_2_ incubator, before staining actin and then visualizing fluorescent-gelatin degradation (dark). The dark-spotted ECM-degraded area from images more than 5 random areas were saved and a representative set was shown. Data represent three independent experiments. **Figure S4.** Chang-TGFβ1 cells transfected with shRNA against TM4SF5 (shTM4SF5) or CD151 (shCD151) without or with TM4SF5 or CD151 cDNA plasmids were reseeded on coverglasses precoated with Oregon Green 488-conjugated gelatin and incubated for 18 h in a CO_2_ incubator, before staining actin and then visualizing fluorescent-gelatin degradation (dark). The dark-spotted ECM-degraded area from images more than 5 random areas were saved and a representative set was shown. Data represent three independent experiments.
(DOC)
