# Correction: Correlations between Transmembrane 4 L6 Family Member 5 (TM4SF5), CD151, and CD63 in Liver Fibrotic Phenotypes and Hepatic Migration and Invasive Capacities

**DOI:** 10.1371/journal.pone.0110148

**Published:** 2014-10-02

**Authors:** The PLOS ONE Staff

The affiliation for the fourteenth author is incorrect. Semi Kim is not affiliated with #4 but with #5 Therapeutic Antibody Research Center, Korea Research Institute of Bioscience and Biotechnology, Daejon, Republic of Korea.
