# Supplementary material for: Correlations between Transmembrane 4 L6 Family Member 5 (TM4SF5), CD151, and CD63 in Liver Fibrotic Phenotypes and Hepatic Migration and Invasive Capacities
Source: PLoS One. 2014 Jul 17;9(7):e102817. doi: 10.1371/journal.pone.0102817 (PMC4102591; doi:10.1371/journal.pone.0102817)
Supplement: File S1 — Contains the files: Figure S1. Transwell migration analysis was performed for Chang-TGFβ1 cells transfected with the indicated shRNAs or plasmids. The bottom chamber was filled with 10% FBS/DMEM-H and cells were loaded to upper chamber. After 18 h, cells migrated to the bottom surface of the filter was stained and imaged. Representative images more than at least 5 images were considered for counting of migrated cells in each experimental condition. Images of a representative experimental set were shown for each experimental condition. Data represent three independent experiments. Figure S2. Transwell migration analysis was performed for Chang-TGFβ1 cells transfected with the indicated shRNAs or plasmids. The bottom chamber was filled with 10% FBS/DMEM-H and cells were loaded to upper chamber. After 18 h, cells migrated to the bottom surface of the filter was stained and imaged. Representative images more than at least 5 images were considered for counting of migrated cells in each experimental condition. Images of a representative experimental set were shown for each experimental condition. Data represent three independent experiments. Figure S3. Chang-TGFβ1 cells transfected with shRNA against TM4SF5 (shTM4SF5) or CD151 (shCD151) were reseeded on coverglasses precoated with Oregon Green® 488-conjugated gelatin and incubated for 18 h in a CO2 incubator, before staining actin and then visualizing fluorescent-gelatin degradation (dark). The dark-spotted ECM-degraded area from images more than 5 random areas were saved and a representative set was shown. Data represent three independent experiments. Figure S4. Chang-TGFβ1 cells transfected with shRNA against TM4SF5 (shTM4SF5) or CD151 (shCD151) without or with TM4SF5 or CD151 cDNA plasmids were reseeded on coverglasses precoated with Oregon Green 488-conjugated gelatin and incubated for 18 h in a CO2 incubator, before staining actin and then visualizing fluorescent-gelatin degradation (dark). The dark-spotted ECM-degra [file pone.0102817.s001.doc]

**Correlations between Transmembrane 4 L6 Family Member 5 (TM4SF5), CD151, and CD63 in liver fibrotic phenotypes and hepatic migration and invasive capacities**

Minkyung Kang^1,2^, Jihye Ryu^2^, Doohyung Lee^2^, Mi-Sook Lee^2^, Hye-Jin Kim^2^, Seo Hee Nam^3^, Haeng Eun Song^2^, Jungeun Choi^3^, Gyu-Ho Lee^2^, Tai Young Kim^2^, Hansoo Lee^4^, Sang Jick Kim^5^, Sang-Kyu Ye^1^, Semi Kim^4^, and Jung Weon Lee^2,3,6^

**Figure S1 in File S1**


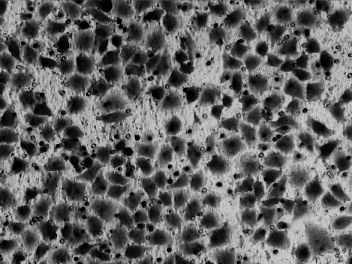

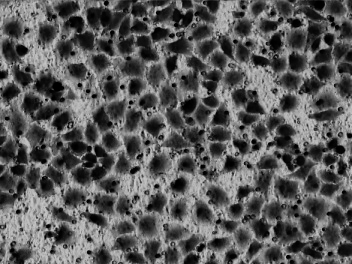


**shControl**

**shCD151**

**shTM4SF5**

**shCD151/**

**shTM4SF5**


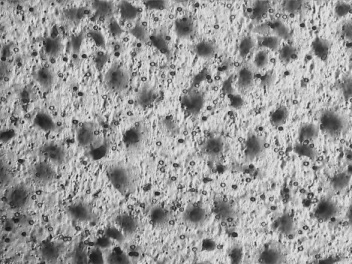

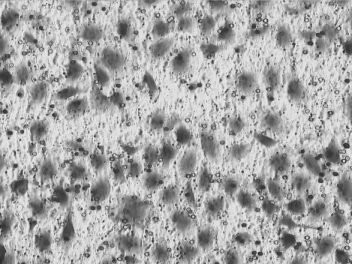

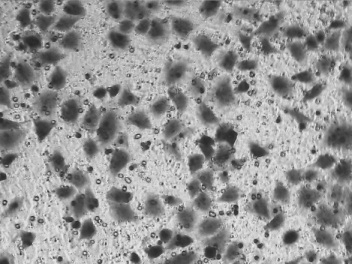

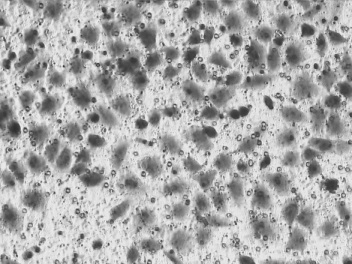

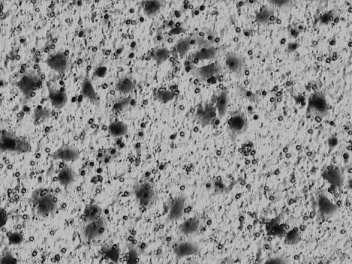

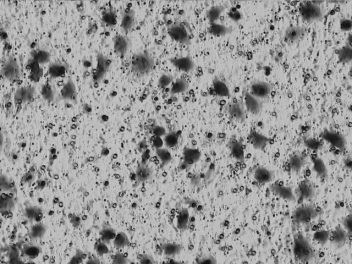


**S1**

Figure S1 in File S1. Transwell migration analysis was performed for Chang-TGFβ1 cells transfected with the indicated shRNAs or plasmids. The bottom chamber was filled with 10% FBS/DMEM-H and cells were loaded to upper chamber. After 18 h, cells migrated to the bottom surface of the filter was stained and imaged. Representative images more than at least 5 images were considered for counting of migrated cells in each experimental condition. Images of a representative experimental set were shown for each experimental condition. Data represent three independent experiments.

**Figure S2 in File S1**


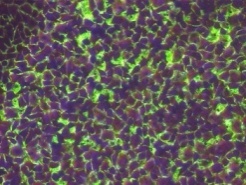

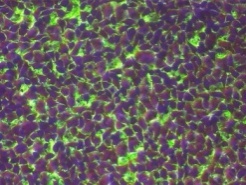


**shControl**


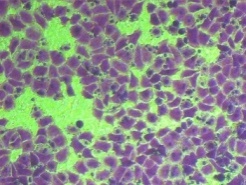

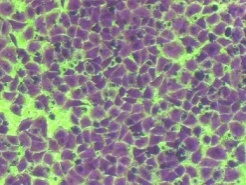

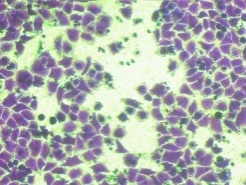

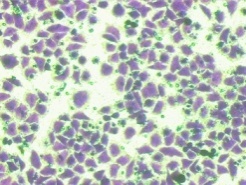


**shCD151**

**/TM4SF5**

**shTM4SF5**

**/CD151**


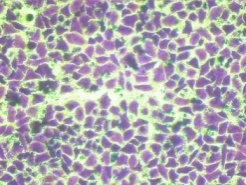

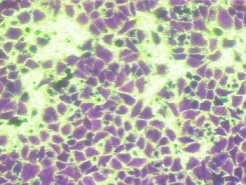


**shCD151**

**shTM4SF5**


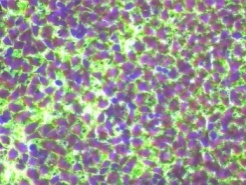

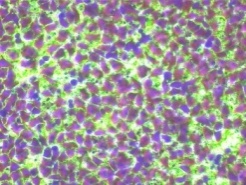


**S2**

Figure S2 in File S1. Transwell migration analysis was performed for Chang-TGFβ1 cells transfected with the indicated shRNAs or plasmids. The bottom chamber was filled with 10% FBS/DMEM-H and cells were loaded to upper chamber. After 18 h, cells migrated to the bottom surface of the filter was stained and imaged. Representative images more than at least 5 images were considered for counting of migrated cells in each experimental condition. Images of a representative experimental set were shown for each experimental condition. Data represent three independent experiments.

**Figure S3 in File S1**

**
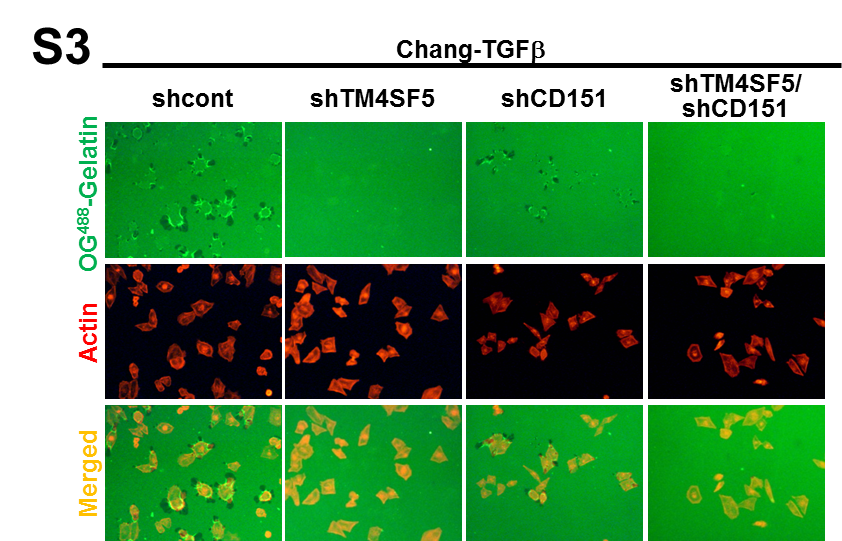
**

Figure S3 in File S1. Chang-TGFβ1 cells transfected with shRNA against TM4SF5 (shTM4SF5) or CD151 (shCD151) were reseeded on coverglasses precoated with Oregon Green® 488-conjugated gelatin and incubated for 18 h in a CO_2_ incubator, before staining actin and then visualizing fluorescent-gelatin degradation (dark). The dark-spotted ECM-degraded area from images more than 5 random areas were saved and a representative set was shown. Data represent three independent experiments.

**Figure S4 in File S1**

**
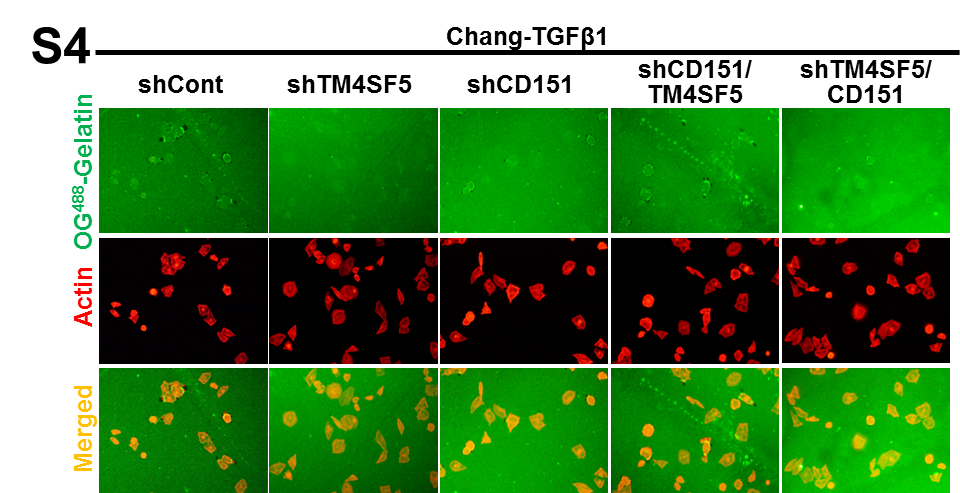
**

Figure S4 in File S1. Chang-TGFβ1 cells transfected with shRNA against TM4SF5 (shTM4SF5) or CD151 (shCD151) without or with TM4SF5 or CD151 cDNA plasmids were reseeded on coverglasses precoated with Oregon Green 488-conjugated gelatin and incubated for 18 h in a CO_2_ incubator, before staining actin and then visualizing fluorescent-gelatin degradation (dark). The dark-spotted ECM-degraded area from images more than 5 random areas were saved and a representative set was shown. Data represent three independent experiments.
